# Mitochondrial Homeostasis and Signaling in Parkinson’s Disease

**DOI:** 10.3389/fnagi.2020.00100

**Published:** 2020-04-21

**Authors:** Antonella Scorziello, Domenica Borzacchiello, Maria Jose Sisalli, Rossana Di Martino, Micaela Morelli, Antonio Feliciello

**Affiliations:** ^1^Division of Pharmacology, Department of Neuroscience, Reproductive and Dentistry Sciences, University of Naples Federico II, Naples, Italy; ^2^Department of Molecular Medicine and Medical Biotechnologies, University of Naples Federico II, Naples, Italy; ^3^Department of Biomedical Sciences, Section of Neuropsychopharmacology, University of Cagliari, Cagliari, Italy

**Keywords:** mitocondria, Parkinson’s disease, cAMP, PKA, AKAP

## Abstract

The loss of dopaminergic (DA) neurons in the *substantia nigra* leads to a progressive, long-term decline of movement and other non-motor deficits. The symptoms of Parkinson’s disease (PD) often appear later in the course of the disease, when most of the functional dopaminergic neurons have been lost. The late onset of the disease, the severity of the illness, and its impact on the global health system demand earlier diagnosis and better targeted therapy. PD etiology and pathogenesis are largely unknown. There are mutations in genes that have been linked to PD and, from these complex phenotypes, mitochondrial dysfunction emerged as central in the pathogenesis and evolution of PD. In fact, several PD-associated genes negatively impact on mitochondria physiology, supporting the notion that dysregulation of mitochondrial signaling and homeostasis is pathogenically relevant. Derangement of mitochondrial homeostatic controls can lead to oxidative stress and neuronal cell death. Restoring deranged signaling cascades to and from mitochondria in PD neurons may then represent a viable opportunity to reset energy metabolism and delay the death of dopaminergic neurons. Here, we will highlight the relevance of dysfunctional mitochondrial homeostasis and signaling in PD, the molecular mechanisms involved, and potential therapeutic approaches to restore mitochondrial activities in damaged neurons.

## Introduction

Mitochondria are the powerhouses of the cell and play an essential role in different metabolic pathways. Derangement of mitochondrial activities have been placed at the center stage of neurodegenerative disorders (Chan, 2020). Efforts have been made to identify the molecular mechanisms governing mitochondrial functions and test the impact of genetic mutations and environmental toxins on mitochondrial activities and cell survival (Carlucci et al., 2008b; Herst et al., 2017).

This is especially important in neurons where alterations of mitochondrial bioenergetics and calcium homeostasis may severely affect synaptic activity and neuronal survival. Parkinson’s disease (PD) is a common neurodegenerative disorder worldwide, characterized by dysfunctional mitochondria and selective loss of nigro-striatal dopaminergic neurons Lin et al., 2019). Restoring mitochondrial function or preventing mitochondrial damage in a reasonable time-window from the disease onset represents a valuable and effective strategy for PD treatment (Iannielli et al., 2018; Sorrentino et al., 2018). Signaling pathways play a major role in mitochondrial activities, including oxidative phosphorylation, cell survival, metabolism, intracellular calcium homeostasis, organelle biogenesis/turnover, and dynamics. Distinct families of protein kinases/phosphatases have been identified as resident of mitochondrial compartments [mitochondrial matrix, intermembrane space, and outer mitochondrial membrane (OMM)]. In response to ligand stimulation, these enzymes catalyze phosphorylation/dephosphorylation events on a variety of mitochondrial substrates, exerting major effects on the organelle physiology. Deregulation of each component of this control mechanism can lead to oxidative stress and mitochondrial damage (Nguyen et al., 2019; Wang et al., 2020). Dysfunctional mitochondria can be eliminated through the autophagy machinery, a process called mitophagy. This is a genetically conserved, homeostatic control mechanism critical for maintaining proper biological functions and cell survival. The execution of mitophagy requires concomitant activation of autophagy and the selective recognition of damaged mitochondria by the autophagosomes. By removing damaged organelles, mitophagy buffers excessive oxidative stress and prevents cell death (Hou et al., 2019). Germline mutations of genes controlling key steps of mitophagy have been identified in PD patients and causally linked to the disease (Harper et al., 2018; Hou et al., 2019). This suggests that restoring mitophagy or modulating the signaling pathways converging on mitophagy can override apoptotic signals in PD neurons.

cAMP is a highly conserved second messenger that is produced by adenylate cyclase in response to the stimulation of G protein-coupled receptors (GPCRs) by hormones or neutransmitters. The principal downstream effector of cAMP is protein kinase A (PKA; Ahuja et al., 2019). Localization of PKA at distinct intracellular compartments by A-Kinase Anchor Proteins (AKAPs) locally modulates key biological activities (Feliciello et al., 2001; Merrill and Strack, 2014; Rinaldi et al., 2016, 2017, 2018). Mitochondrial AKAPs have been isolated and functionally characterized (Merrill and Strack, 2014; Rinaldi et al., 2018). AKAP•PKA complexes assembled on mitochondria support oxidative phosphorylation, metabolism, calcium homeostasis, cell survival, mitophagy, and global protein synthesis (Carlucci et al., 2008b; Rinaldi et al., 2018). Interestingly, upregulation of cAMP signaling to the organelle by AKAPs or pharmacological interference with mitochondria-generated apoptotic pathways reversed oxidative stress, mitochondrial dysfunction, and loss of dopaminergic neurons in PD models (Dagda et al., 2011; Kostic et al., 2015). These studies support the concept that local modulation of signaling cascades at mitochondria constitutes a novel, promising therapeutic strategy for PD.

Here, we will discuss the role of deranged mitochondrial signaling in the development and progression of PD and will provide deeper insight into potential therapeutic strategies to overcome the oxidative damage and dopaminergic cell loss.

## Mitochondrial Quality Control Mechanisms in Healthy and PD Neurons

The physiological clearance of damaged mitochondria by the authophagy machinery, also known as mitophagy, represents an important homeostatic mechanism that assists in the elimination of otherwise altered organelles (Hou et al., 2019; Boyman et al., 2020). Preventing or interfering with the mitophagic removal of damaged mitochondria may lead to oxidative stress and irreversible apoptotic cell death, as it occurs in dopaminergic neurons in PD (Hou et al., 2019). Autophagy is an essential catabolic mechanism that delivers misfolded proteins and damaged organelles to lysosomes for degradation. Autophagy dysfunction has been considered a relevant pathogenetic mechanism leading to neurodegeneration in PD, since a decreased expression of proteins involved in the autophagy–lysosome pathway and an impairment in the activity of lysosomal enzymes have been described in PD patients (Moors et al., 2017). Indeed, the expression of the lysosomal-associated membrane protein 1 (LAMP1), a glycoprotein responsible for lysosomal integrity, is reduced in the Substantia Nigra pars compacta (SNpc) of PD patients. Similarly, the activity of the lysosomal β-glucocerebrosidase (GCase) is downregulated in different brain regions and also in the cerebrospinal fluid of PD patients (Rocha et al., 2015). Moreover, the expression of transcription factor EB (TFEB), the master regulator of lysosomal biogenesis and autophagy, is significantly decreased in SNpc dopaminergic neurons of PD patients and its staining co-localizes with Lewy bodies, a neuropathological hallmark of PD (Decressac et al., 2013). Interestingly, genetic mutations associated with increased risks for developing PD, like α-synuclein, LRRK2, beta-glucosidase 1 (GBA1; lysosomal glucocerebrosidase), Parkin, PTEN-induced kinase 1 (PINK1), DJ-1, Fbxo7, and VPS35, encode for components of the autophagy–lysosome pathway, thus confirming a tight link between the autophagy dysfunction and PD (Gan-Or et al., 2015; Verstraeten et al., 2015; Przedborski, 2017). Of relevance are the observations that either mutations of genes linked to familial forms of PD or environmental factors responsible for sporadic forms of PD both induce mitochondrial dysfunction. At synapses, mitochondria play a major role in energy metabolism, calcium homeostasis, signaling, and neurotransmitter release. Thus, deregulation of mitochondrial activities and homeostasis contributes to synaptic impairment, as in occurs in PD (Nguyen et al., 2019).

In healthy mitochondria, the protein kinase PINK1 accumulates within the mitochondrial matrix (Huang et al., 2017). This process requires the coordinated action of the translocase of the inner membrane complex (TIM) in conjunction with the translocase of the outer membrane complex (TOM), as well as the activity of several proteases and translocases. At the end of these events, a proteolytically processed form of PINK1 is released from mitochondria to the cytosol, where eventually it undergoes degradation. Depolarization of the mitochondrial permeability transition pore (PTP) leads to the accumulation of full length PINK1 at the OMM, where it forms a macromolecular complex with TOM proteins (Koyano et al., 2019). Here, PINK1 phosphorylates ubiquitin moieties and promotes phospho-Ub (pUb)-mediated recruitment of the E3 ligase Parkin to the OMM and its activation by conformational changes. Once activated, Parkin ubiquitylates in induced neurons several mitochondrial proteins, including mitofusins 1/2 (Mfn1/2), two homolog proteins involved in the mitochondria dynamics (fusion/fission), and the voltage-dependent anion channel-1 (VDAC1), a major regulator of the exchange of ions and small molecules across the OMM (Ordureau et al., 2018). Ubiquitinated Mfn2 recruits the p97 segregase. The Mfn2/p97 complex is then released from OMM in the cytosol where Mfn2 is degraded through the proteasome. Downregulation of Mfn2 induces loss of the endoplasmic reticulum-mitochondria contact sites and membrane fusion activity, which culminates in mitochondrial fission (Tanaka et al., 2010). Concomitantly, ubiquitylation of VDAC1 at OMM by Parkin1 recruits the autophagy receptor optineurin (OPTN), which in turn induces autophagosome formation and mitophagy (Ordureau et al., 2018). Alternatively, vesicles containing mitochondrial damaged proteins (mitochondria-derived vesicles, MDVs) can be eliminated through the selective autophagy machinery (McLelland et al., 2014; Matheoud et al., 2016). In both cases, the mitophagy system plays a central role in the homeostatic control of mitochondrial clearance.

A positive interplay between PINK1 and mitochondrial trafficking along neuronal dendrites and axons is critical for the homeostatic clearance of damaged mitochondria. Under stress conditions, recruitment of PINK1 to mitochondria promotes phosphorylation and Parkin-mediated proteolysis of Miro, a component of the primary motor/adaptor complex that anchors kinesin motor to mitochondria and is an essential regulator of mitochondrial transport. Removal of Miro induces mitochondrial stalling at cell periphery and their consequent clearance through the mitophagy system (Weihofen et al., 2009; Wang et al., 2011). PINK1 also regulates mitochondrial trafficking through PINK1-induced recruitment of PKA to mitochondrial AKAP1. Here, AKAP1-bound PKA phosphorylates Miro2, another member of the Miro protein family, and promotes mitochondrial trafficking. Following oxidative stress, PINK1 uncouples mitochondrial PKA signaling and induces mitophagy of damaged mitochondria. Neurons lacking PINK1 show impaired mitochondrial trafficking and dendrite outgrowth. These defects can be rescued by overexpressing AKAP1, suggesting that PKAs and PINK1 converge on mitochondria to control the mitochondrial trafficking and quality control system (Pryde et al., 2016; Das Banerjee et al., 2017). Very recently, it emerged that Parkin-mediated ubiquitylation of membrane associated ring-CH-type finger 5 (MITOL/March5), an E3 ligase localized at OMM, promotes the ATPase valosin-containing protein (VCP)/p97-dependent extraction and translocation of MITOL/March5 from mitochondria membrane to peroxisomes through a mechanism involving the peroxisomal biogenesis factors (Pex3/16). This constitutes a fine sensor mechanism that, in response to oxidative stress and mitochondria damage, allows mitochondrial proteins to escape the mitophagy process and regulate the activity of other organelles (Koyano et al., 2019). Derangement of this quality control system, as a consequence of mutations involving PINK1, Parkin and other genes working on the same regulatory pathway may lead to the accumulation of damaged mitochondria, oxidative stress, and neuronal cell loss.

The deubiquitination system tightly counteracts the PINK1-Parkin pathway and contributes to mitochondrial homeostasis. Thus, the deubiquitinase USP30 is localized at the mitochondrial compartment where it deubiquitinates Parkin substrates. By removing the ubiquitin moieties from proteins, USP30 exerts negative effects on Parkin-mediated mitophagy. Interestingly, genetic downregulation of USP30 in flies carrying PD-linked mutations of Parkin or PINK1 reversed the mitophagy defects and restored mitochondrial integrity. Moreover, USP30 knockdown waived the dopaminergic defects in the treated flies, including motor defects and organismal survival (Bingol et al., 2014). This exemplifies a sophisticated mechanism operating in mitochondria that finely regulates the ubiquitin system to control the homeostatic processes underlying metabolism, respiration, and oxidative stress.

## Mitochondrial Metabolism, Dynamics and Trafficking in PD

Accumulation of α-synuclein and mitochondrial dysfunction has also been observed in neurons subjected to oxidative stress. Under these conditions, high levels of oxidized dopamine impair glucocerebrosidase enzymatic activity and promotes mitochondrial/lysosomal dysfunction. These effects can be reproduced, increasing the levels of dopamine in the mouse brain or overexpressing α-synuclein, implying that the interplay between mitochondria and lysosomes are crucial for the maintenance of cell homeostasis (Burbulla et al., 2017). The transition from monomeric to beta sheet-rich oligomers of α-synyclein leads to its accumulation on mitochondria, affecting the organelles morphology and the oxidative phosphorylation. Specifically, α-synuclein oligomers interact with and oxidize components of the ATP synthase complex, affecting the respiratory chain activity. Moreover, oligomers strongly induce mitochondrial lipid peroxidation, leading to mitochondrial PTP opening, activation of the apoptotic pathway, and neuronal cell death, as it occurs in PD (Ludtmann et al., 2018). Cardiolipin is an essential phospholipid of the mitochondrial membranes and has a major role in key mitochondrial processes, such as oxidative phosphorylation and energy conversion (Dudek, 2017). In α-synuclein expressing neurons, accumulation of α-synuclein on mitochondria has been linked to increased exposure of cardiolipin to the OMM, a sophisticated mechanism adopted by the cell to refold α-synuclein fibrils and, thus, counteract the accretion of Levi’s bodies. However, chronic exposure of mitochondria to cardiolipin promotes recruitment of components of the autophagy machinery which culminates in excessive elimination of mitochondria with consequent neuronal cell loss (Ryan et al., 2018).

Mutations of leucine-rich repeat kinase 2 (LRRK2) are the most common monogenic causes of familial and sporadic forms of PD (Tolosa et al., 2020). LRRK2 is a direct substrate of PKA. Thus, PKA phosphorylation at its Ras of complex proteins (ROC) GTPase domain (RASpS_1444_) rapidly promotes recruitment of 14.3.3 proteins to LRRK2. Binding to 14.3.3 significantly reduces LRRK2 kinase activity. Mutations at the ROC motif in PD patients dramatically affect LRRK2 phosphorylation by PKA, increasing its kinase activity. In striatal neurons, LRRK2 can also act as an AKAP-like protein targeting cAMP-dependent protein kinase type II beta regulatory subunit (PKARIIβ) at specific subcellular compartments. During synaptogenesis, or in response to dopamine receptor D1 (Drd1) activation, LRRK2 spatially restrains PKA activity in the striatal projection neurons. Loss of LRRK2 activity or a PD-linked missense mutation within the RIIβ-binding domain (R1441C) of LRRK2 aberrantly induced a widespread, uncontrolled PKA phosphorylation of cellular substrates, including glutamate ionotropic receptor AMPA type subunit 1 (GluR1; Muda et al., 2014; Parisiadou et al., 2014). These findings indicate the existence of a mutual control of LRRK2 and PKA activities within dopaminergic neurons, and derangement of this control circuitry may have a pathogenetic relevance for PD. LRRK2 also controls the activity of a member of the RAS oncogene family (RAB10) at mitochondrial sites. Under oxidative stress, LRRK2 phosphorylates RAB10 and induces its accumulation on depolarized mitochondria through a mechanism involving PINK1 and Parkin. Mitochondria-bound RAB10 recruits the autophagy receptor OPTN and induces mitophagy. In PD neurons carrying the most common LRRK2 mutations (G2019S and R1441C), RAB10 phosphorylation at threonine 73 is enhanced, and this correlates with a marked reduction of mitochondrial activities and autophagic clearance (Wauters et al., 2019). These findings support a model whereby LRRK2, PINK1, and Parkin in dopaminergic neurons are aligned on the same mitochondrial pathway, and mutations of each of these key elements can cause PD.

Vacuolar protein sorting-associated protein 35 (VPS35), a gene product of the vacuolar protein sorting gene family and component of the retromer complex, controls the retrograde transport of proteins from endosomes to trans-Golgi networks. Mutations of VPS35 have been identified in PD patients. Functional analysis revealed that expression of mutant VPS35 induced mitochondrial fragmentation and increased death of dopaminergic neurons *in vitro* and in the substantia nigra, as well as in fibroblasts isolated from PD patients carrying the VPS35 (D620N) mutation. At a mechanistic level, mutant VPS35 interacts with mitochondrial dynamin-like protein 1 (DLP1) and enhances the proteolytic turnover of DLP1 complexes trafficking from mitochondria to lysosomes, increasing mitochondrial fission and organelle dysfunction. Interfering with the mitochondrial fission reverses the effects of mutant VPS35 in cultured neurons (Wang et al., 2016).

Genetic mutations of PLA2G6 (PARK14), a Ca-independent phospholipase A2 group 6, have been causally linked to sporadic cases of PD. Accordingly, the loss of the fly homolog of PLA2G6, iPLA2-VIA, reduces lifespan and affects synaptic transmission, promoting neurodegeneration. Mutations of PLA2G6 affects its ability to increase the activity of retromer VPS35 and VPS26 proteins, leading to the accumulation of ceramide, an intermediate sphingolipid and essential component of membranes or cellular organelles. High levels of ceramide, in turn, affect membrane fluidity and mitochondrial activities, leading to neurodegeneration. Accordingly, pharmacologically reducing the levels of ceramide alleviates lysosomal stress and mitochondrial dysfunction, and suppresses neurodegeneration, suggesting that disruption of ceramide metabolism may affect endolysosomal and mitochondrial function. Similar effects were observed in neurons lacking VPS35 or VPS26, or in cells overexpressing α-synuclein (Lin et al., 2018, 2019).

Coiled-coil-helix-coiled-coil-helix domain containing 2 (CHCHD2) belongs to a class of eukaryotic transcription factors containing four cysteines spaced ten residues apart from one another (Baughman et al., 2009). CHCHD2 contains a mitochondrial targeting sequence at its N-terminus that localizes the protein within the intermembrane compartment of the organelle. CHCHD2 interacts with cytochrome c and with members of the Bax inhibitor-1 superfamily, positively impacting on respiration and cell survival. Following mitochondrial stress, CHCHD2 translocates to the nucleus and promotes the mitochondrial respiratory gene expression. This is a conserved adaptive regulatory system that cells and neurons adopt to cope with stress conditions (Imai, 2020). Mutations of the CHCHD2 gene have been identified in familial cases of PD (Funayama et al., 2015). Functional and structural analyses of the mutation in flies revealed that loss of CHCHD2 causes abnormal mitochondrial matrix structures and compromises the efficiency of the respiratory chain, leading to oxidative stress, dopaminergic neuronal loss, and PD-like motor defects. This phenotype could be rescued by overexpressing the translation inhibitor 4E-BP1 (eukaryotic translation initiation factor 4E-binding protein 1), a suppressor of cap-dependent protein translation and a positive regulator of neuronal survival (Meng et al., 2017).

PGAM family member 5, mitochondrial serine/threonine protein phosphatase (PGAM5), is a serine/threonine protein phosphatase involved in essential aspects of mitochondrial homeostasis. In particular, PGAM5 interacts with and dephosphorylates several mitochondrial substrates, including apoptosis inducing factor (AIF), FUN14 domain containing 1 (FUNDC1), and dynamin related protein 1 (Drp1), regulating metabolism and mitophagic cell death in response to oxidative stress and mitochondrial fission (Wang et al., 2012; Chen et al., 2014; Lenhausen et al., 2016). PGAM5 is also required for the accumulation of PINK1 on damaged mitochondria and its deficiency prevents PINK1-mediated mitophagy, promoting a PD-like phenotype (Lu et al., 2014). A similar mechanism involves the phosphatase and tensin homolog L homeolog (PTEN-L), a newly identified isoform of PTEN. When localized on the OMM, PTEN-L dephosphorylates the phosphoSer65-ubiquitin molecule, counteracting the PINK1-mediated phosphorylation of ubiquitin. In doing so, PTEN-L antagonizes the PINK1-mediated mithophagy, leading to the accumulation of damaged mitochondria and cell death. This finding suggests that PTEN-L-mediated regulation of the mitochondrial clearance system represents an additional Achilles heel in the pathogenesis of PD (Wang et al., 2018).

## Mitochondrial Dysfunction, Inflammatory Responses and Immune System in PD

Evidence indicates the existence of a pathogenetic link between inflammatory responses, the immune system, and the onset and progression of PD. High levels of circulating inflammatory cytokines, T cell infiltration, and glial cell reactions represent common features in several variants of PD, and also in mouse models of PD (Labzin et al., 2018; Caggiu et al., 2019; Kumar, 2019). Mitochondrial damage triggers inflammatory responses that may cause selective loss of dopaminergic neurons, giving rise to a PD phenotype. By eliminating damaged mitochondria, mitophagy counteracts the oxidative stress and the release of inflammatory cytokines (Martins et al., 2018). Mutations affecting the activity of Parkin or PINK1, the two major controllers of mitochondrial homeostasis, induce the accumulation of damaged mitochondria, enhancing the oxidative stress and promoting the release of damage-associated molecular patterns (DAMPs; Sliter et al., 2018). DAMPs are host biomolecules that initiate and sustain noninfectious inflammatory responses, promoting the release of inflammatory citokines (interleukin 6, tumor necrosis factor, interleukin 1 beta, and interferon gamma). The effects of DAMPs are mediated by STING (stimulator of interferon genes), a signaling protein that acts as a major regulator of the innate immune pathway, controlling the expression of numerous host defence genes and pro-inflammatory cytokines. Interfering with STING activity supported dopaminergic neuronal survival under oxidative stress or Parkin deficiency and restored motor activity (Sliter et al., 2018).

Deregulation of the immune system has been causally linked to motor deficit and PD-like phenotypes. Thus, genetic inactivation of INF-β (Interferon beta), a cytokine of the interferon family, induced a spontaneous neurodegenerative phenotype with motor deficits and learning impairment, even in the absence of disease-associated genetic mutations. Mice lacking INF-β show accumulation of α-synuclein-containing Lewy bodies in the brain, reduced mitophagy, accretion of senescent and damaged mitochondria, and loss of dopaminergic neurons within the nigro-striatal area. Reintroduction of exogenous INF-β rescued most of the neuronal alterations, restoring mitophagy, neurite growth and branching, and elimination of α-synuclein aggregates. Moreover, in mouse models of familial PD, lentiviral expression of INF-β prevented the loss of dopaminergic neurons, supporting a safeguarding role of the immune system in healthy neurons (Ejlerskov et al., 2015).

A link between the PINK1-mediated mitochondrial clearance system and the innate immunity in PD has recently been discovered. Thus, intestinal infections with Gram-negative bacteria in PINK1^−/−^ mice induce the generation of cytotoxic CD8^+^ T cells directed against mitochondrial antigens, causing selective loss of striatal dopaminergic structures and t functional motor defects. This phenotype can be rescued by L-3,4-dioxyphenylalanine (L-DOPA) treatment. Thus, PINK1-Parkin system operates as a repressor of the adaptive immune system and safeguard mechanism for healthy mitochondria. In the presence of PINK1 mutations, bacterial infections may trigger an autoimmune response against mitochondrial antigens, inducing loss of dopaminergic neurons and PD (Matheoud et al., 2019).

## Mitochondrial Calcium Homeostasis in PD

Dysregulation in calcium (Ca^2+^) homeostasis participates in the pathogenesis of PD (Surmeier et al., 2011). Thus, the mechanisms regulating Ca^2+^ handling in dopaminergic neurons and the role exerted by PD-related proteins in the control of intracellular Ca^2+^ homeostasis have been highlighted (Cali et al., 2011, 2012). Mitochondria play a key role in the control of cytosolic Ca^2+^ buffering and cellular metabolism. Accordingly, deranged mitochondrial calcium homeostasis has been pathogenically linked to neurodegeneration occurring in PD (Bose and Beal, 2016; Verma et al., 2017). The maintenance of mitochondrial calcium concentrations within physiological range, necessary for neurons to regulate erobic ATP production and synaptic transmission and excitability, is strictly dependent on the synchronized activity of specific transporters and channels whose identity has been recently determined (De Stefani et al., 2011; Palty et al., 2012). Calcium enters into mitochondria through the VDAC channel localized on the outer membrane (Tan and Colombini, 2007), and is transported across the intermembrane space and the inner membrane into the matrix by the mitochondrial calcium uniporter (MCU) and its docking/regulatory proteins MICU1/MICU2 (Tan and Colombini, 2007). Similarly, calcium is exported from the mitochondrial matrix through LETM1 or the Na^+^/Ca^2+^exchanger NCLX, in exchange for H^+^ or Na^+^/Li^+^, respectively and, then, across the outer membrane through VDAC and the isoform 3 of the sodium calcium exchanger (NCX3; Tan and Colombini, 2007; Scorziello et al., 2013; Anderson et al., 2019). In PD neurons, the activation of mitochondrial Na^+^/Ca^2+^ exchanger (mNCX) is the primary mechanism by which mitochondrial calcium concentrations [Ca^2+^]_m_ is returned to the cytoplasm, and therefore it is critical to a multitude of Ca^2+^-dependent processes including neurotransmitters release, synaptic plasticity, bioenergetics and mitochondrial nitric oxide (NO), and reactive oxygen species (ROS) production (Castaldo et al., 2009; Cali et al., 2013). In the absence of PINK1, mNCX activity was severely impaired, leading to mitochondrial calcium overload, PTP opening, and cell death (Gandhi et al., 2009). Moreover, in human dopaminergic neurons, plasmalemmal NCX2 and NCX3 contribute to mitochondrial Na^+^/Ca^2+^ exchange and may act downstream of PINK1 in the prevention of neurodegeneration by [Ca^2+^]_m_ overload (Wood-Kaczmar et al., 2013). In primary mesencephalic neurons from A53T α-synuclein transgenic mice embryos, downregulation of NCX3 levels is linked to mitochondrial depolarization and mitochondrial calcium increase, compared to wild type neurons, suggesting that mitochondrial dysfunction in PD is linked to mitochondrial Ca^2+^ mishandling (Cali et al., 2013; Sirabella et al., 2018). However, the crucial question is whether the disease begins with the impairment of mitochondrial dysfunction, or whether this is a consequence of derangement of other dopaminergic neuronal activities. Interestingly, SNpc dopaminergic neurons are autonomously active since they generate action potentials in the absence of conventional synaptic input (Grace and Bunney, 1983). Due to this pace-making activity, DA neurons are more prone to frequent transients of calcium which are necessary to allow DA release in the connected brain areas (e.g., the striatum) and consequently to the control of coordinated movements. In this scenario, the mechanisms involved in the maintenance of ionic homeostasis are forced to work under stressful conditions, including mitochondria whose calcium buffering activity is boosted to maintain cytosolic calcium within physiological ranges. Indeed, the sustained entry of Ca^2+^ into mitochondria stimulates the ATP-dependent pumps responsible for keeping Ca^2+^ concentration low, altering mitochondrial oxidative phosphorylation (Surmeier et al., 2011). As a consequence, overproduction of superoxide ions leads to DNA and proteins oxidation. This metabolic stress exacerbates the aging-related decline of mitochondrial function, resulting in decreased energy supply and increased sensitivity to cell death (Guzman et al., 2010). Based on this evidence, Ca^2+^ homeostasis can be considered as an early feature of PD rather than a consequence of neuronal derangements. In particular, Ca^2+^ entry during the pace-making activity of SNpc neurons can amplify the effects of genetic mutations or of the environmental factors, thus speeding forward neuronal aging and death. Interestingly, ventral tegmental area neurons, which are more resistant to neurodegeneration, show a slow pace-making activity and do not manifest Ca^2+^ oscillations (Chan et al., 2007). Conversely, non-mesencephalic neurons, e.g., olfactory and some hypothalamic neurons, are characterized by spontaneously high activity associated with conspicuous Ca^2+^ currents and low buffering capacity, and undergo degeneration during PD progression (Surmeier and Schumacker, 2013).

The link between calcium homeostasis and mitochondrial dysfunction in PD progression is further supported by the finding that α-synuclein, the main component of Lewy bodies to be considered a neuropathological hallmark of PD, might exert its toxicity through the engagement of the Ca^2+^ homeostatic machinery. However, the molecular mechanisms responsible for these effects are still unknown. One possibility is that α-synuclein, as aggregation-prone protein, forms fibrillary oligomers that enhance plasma membrane permeability to Ca^2+^influx, inducing an intracellular Ca^2+^ overload and consequent neuronal toxicity (Danzer et al., 2007; Schmidt, 2012; Tsigelny et al., 2012). Moreover, the increase in intracellular Ca^2+^ may promote α-synuclein aggregation, thus leading to a vicious cycle which further increases intracellular Ca^2+^ levels. Recent studies also demonstrated that α-synuclein associates with mitochondria, and that its accumulation within the organelle was directly related to an increase of intramitochondrial Ca^2+^ levels (Parihar et al., 2008), which in turn led to a rise of NO levels, oxidative damage, and cytochrome c release from mitochondria (Parihar et al., 2009), leading to apoptotic neuronal death. This finding supports the model whereby α-synuclein has a major role in modulating not only the cellular, but also the mitochondrial Ca^2+^ fluxes. Accordingly, in A53T α-synuclein mesencephalic neurons, the rise of mitochondrial calcium concentration is linked to depolarization of mitochondrial membrane (Sirabella et al., 2018).

## Potential Therapeutic Strategies for PD

**Figure 1:**
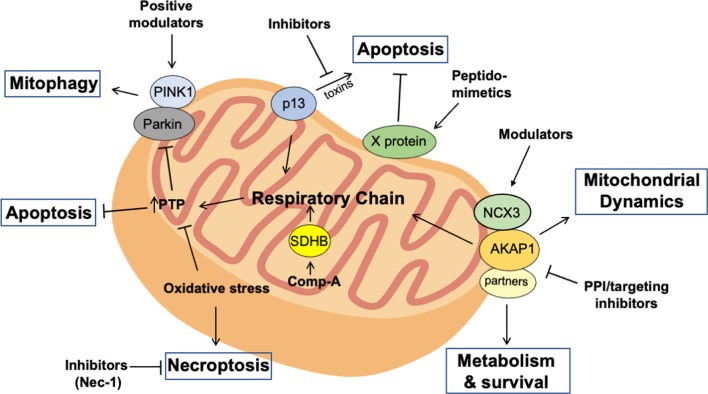
Targeting mitochondria for the treatment of Parkinson’s disease (PD). Distinct experimental approaches can be used to selectively interfere with the mutation-induced deregulation of mitochondrial activities in PD neurons. Thus, deranged mitochondrial cAMP signaling can be restored using protein-protein interaction (PPI)/targeting molecules that support the assembly of A-Kinase Anchor Protein (AKAP) complexes at mitochondria. This would positively impact on cell survival and metabolism of PD neurons. Similarly, peptidomimetics that positively regulate X protein activity at mitochondria have been successfully used to prevent oxidative stress and apoptotic cell death induced by mitochondrial toxins. Similarly, inhibiting p13 or treating with necrosis inhibitors (Nec-1) in toxin-induced or familial forms of PD positively impacts on oxidative metabolism and neuronal survival. Moreover, restoring PINK1-Parkin pathway using selective agonists or preventing ubiquitin-specific protease (USP)-mediated deubiquitination of Parkin substrates with selective USP inhibitors could restore mitophagy in PD neurons, preventing accumulation of damaged mitochondria, oxidative stress, and neuronal apoptosis. Finally, sustaining the activity of respiratory enzymes as succinate dehydrogenase subunit B (SDHB) with chemical drugs represents a promising therapeutic approach to restore oxidative metabolism and mitochondrial permeability transition pore (PTP), preventing apoptotic or necroptotic neuronal loss.

Different molecular approaches have been proposed and experimentally tested for the treatment of PD and PD-like disorders. P13 is a mitochondria-localized protein that plays an important role in mitochondrial respiration. Overexpression of p13 promoted deregulation of mitochondrial activities and induced apoptotic cell death. Conversely, genetic downregulation of p13 alleviated the effects of toxin-induced mitochondrial dysfunction and apoptosis of dopaminergic SH-SY5Y cells. Similarly, heterozygous p13 knockout mice are protected against toxin-induced motor deficits and dopaminergic neuronal loss in the substrantia nigra (Inoue et al., 2018). Inhibition of p13, thus, represents a promising strategy for PD treatment (Figure 1).

Studies on viral infections have been useful for the design of novel approaches for the treatment of neurodegenerative diseases caused by excessive oxidative stress and mitochondrial dysfunction. Borna disease virus (BDV) is a neurotropic RNA virus that infects the brain of several animal species without compromising neuronal activity. The replication of the virus is dependent on the expression of a virus-encoded protein, named X protein. This protein controls RNA synthesis and polymerase complex formation within the nuclear compartment, playing an important role in viral replication. X protein also localizes at mitochondria and protects infected cells from apoptotic cell death, favoring evasion of the virus from host-cell responses and promoting a non-cytolytic viral infection (Poenisch et al., 2009). Interestingly, when expressed in cultured neurons, X protein protects from oxidative stress induced by mitochondrial toxins and prevents mitochondrial fragmentation and cell death. Moreover, in mouse model of PD, X protein protects nigro-striatal domapinergic neurons from toxin-induced neurodegeneration. Similarly, intranasal administration of a cell-permeable peptide designed on the X protein sequence mimicked the effects of the full length protein, protecting PD dopaminergic neurons from mitochondrial stress and apoptosis in toxin-treated mice (Figure 1; Szelechowski et al., 2014).

The identification of molecules that selectively interfere with the mitochondrial apoptotic pathway represents a valuable approach for PD treatment. Several biologically relevant molecules have been isolated by chemical screenings and functionally characterized as novel drugs potentially useful for the treatment of neurodegenerative diseases. Among these, compound A has been shown to have a protective role for dopaminergic neurons in animal models of PD. Thus, compound A specifically and covalently interacts with succinate dehydrogenase subunit B (SDHB) of the complex II of the electron transport chain. In cultured cells, the binding of compound A to SDHB protects the electron transport chain from proapoptotic Bcl-2 homology 3-only protein Bim-induced depolarization of PTP, preventing the release of cytochrome c and consequent activation of the apoptotic cascade. *In vivo*, injection of compound A in the brain of rat models of PD prevented neurotoxicity and loss of dopaminergic neurons (Figure 1; Jiang et al., 2016).

A similar approach has been employed to interfere with the death of pluripotent stem cell (iPSC)-derived neural cells (NPCs) generated from skin fibroblasts of patients carrying PD-associated OPA1 mutations. NPCs show marked mitochondrial dysfunctions, impaired oxidative phosphorylation, and an elevated oxidative stress which causes neuronal necroptosis, a regulated process of necrosis triggered by inflammatory signals. Pharmacological treatment with NEC1s, a specific and potent inhibitor of necroptosis, increased NPCs survival *in vitro* and markedly attenuated toxin-induced nigrostriatal degeneration and mitochondrial dysfunction in mice (Figure 1; Iannielli et al., 2018).

Epoxide hydrolase (sEH) is a soluble enzyme that plays a significant role in inflammation. The expression of this enzyme is increased in post-mortem brain tissues from patients with dementia Lewy bodies (DLB) and in the striatum of mice treated with 1-methyl-4-phenyl-1,2,3,6-tetrahydropyridine (MPTP), a mitochondrial neurotoxin that induces severe PD-like symptoms in humans and primates. Furthermore, the levels of sEH correlated with the phosphorylation of α-synuclein in dopaminergic brain neurons. Metabolites analysis confirmed an increased activity of sEH in the striatum area of MPTP-treated mice. Interestingly, in human Parkin iPSC-derived neurons, the levels of sEH mRNA was increased, compared to healthy controls. Treatment with the 1-(1-Propionylpiperidin-4-yl)-3-(4-(trifluoromethoxy)phenyl)urea (TPPU), a potent sEH inhibitor, prevented the apoptosis in Parkin iPSC-derived dopaminergic neurons, suggesting that sEH represents a valuable target for the treatment of neurodegenerative disorders and PD (Figure 1; Ren et al., 2018).

The conversion of α-synuclein into fibrillar phosphorylated α-synuclein aggregates similar to Lewy bodies has been pathogenically linked to PD. The aggregates undergo the elimination through the autophagy machinery. During this process, a non-fibrillar, phosphorylated α-synuclein species (pα-syn) accumulates within the cells, at the endoplasmic reticulum-mitochondrial interface, where it forms multimeric complexes with components of metabolic pathways (acetyl-CoA carboxylase 1) and regulators of the unfolded protein response (binding immunoglobulin protein, BiP). This leads to a reduction of ATP levels, activation of AMP-activated protein kinase (AMPK), and oxidative stress, which culminates in mitochondrial fragmentation and death of dopaminergic neurons. Pα-syn found in primary neurons seeded on fibrils of recombinant α-syn, and also in the brain of mice treated with recombinant α-syn fibrils and in the brain of PD patients (Grassi et al., 2018). These findings suggest a relevant pathogenetic role of pα-syn species in the development and/or progression of PD, offering a novel therapeutic target for PD treatment.

Transcriptional upregulation of genes encoding for proteins involved in the mitochondrial mass have been used to dampen mitochondrial stress in mouse models of PD. Thus, the cell cycle inhibitor Necdin, when overexpressed in the striatum, promoted mitochondrial biogenesis by preventing the ubiquitin-dependent proteolysis of the transcriptional co-activator, peroxisome proliferator-activated receptor-gamma coactivator-1 alpha (PGC1α). In cortical neurons and in human SH-SY5Y neuroblastoma cells, overexpression of necdin enhanced mitochondrial activities and prevented neuronal degeneration induced by inhibitors of the respiratory chain. Moreover, forced expression of necdin in the substantia nigra *in vivo* prevented the loss of dopaminergic neurons in experimental models of mouse PD. This finding suggests that using molecules that selectively interfere with the ubiquitin-dependent proteolysis of PGC1α could have a therapeutic application in preventing the neuronal loss in course of PD (Hasegawa et al., 2016).

PINK1-Parkin pathway is an important quality control system that efficiently eliminates damaged mitochondria through mitophagy, thus contributing to neuronal fitness and protection against oxidative stress. Mutations affecting one or more members of this regulatory system may expose neurons to mitochondrial stress, which eventually leads to cell death. A therapeutic strategy might target PINK1-Parkin axis with selective agonists or preventing USP30-mediated deubiquitination of Parkin substrates. Treatment with USP30 inhibitors should restore the mitophagy machinery, counteracting oxidative stress and loss of PD neurons (Figure 1).

Targeting compartmentalized cAMP signaling represents a valuable strategy for PD treatment. AKAPs control important aspects of neuronal activity. Disruption of AKAP-regulated targeting of PKA signals affects ion channel activities, neuronal transmission, synaptic plasticity, learning and memory, metabolism, and neuronal survival (McConnell et al., 2009; Kennedy and Scott, 2015; Rinaldi et al., 2015, 2018). The relative abundance in different brain areas, the ability to coordinate different signaling pathways, and the peculiar localization at discrete intracellular sites render AKAPs essential elements for highly specialized brain activities. Manipulating the assembly of AKAP scaffold within neuronal cells by Protein-Protein Interaction (PPI) inhibitors represents a valuable approach in translational medicine. Thus, peptides spanning the amphipathic helical wheel of AKAPs have been used successfully to delocalize PKA from discrete intracellular sites, both in cultured cells and *in vivo* (Wang et al., 2015; Dukic et al., 2017). However, the lack of specificity vs. distinct AKAPs and the off-target effects represent major limitations for the use of PKA displacing molecules. An alternative strategy is the use of PPI disruptors that interfere with the regulation of the AKAP module by disease-activated pathways. In this context, mitochondrial AKAP1 represents a valuable target for the treatment of neurodegenerative diseases (Rinaldi et al., 2018). In the case of ischemic insult in the heart or brain, the E3-ubiquitin ligase Siah2 rapidly accumulates within cells, leading to ubiquitination and proteolysis of AKAP1. Downregulation of AKAP1 reduces mitochondrial respiration and ATP synthesis (Carlucci et al., 2008a). This provides a fine feed-back mechanism of signal attenuation in the presence of limiting amounts of oxygen (Carlucci et al., 2008b). In neurons and in cardiomyocytes, prolonged downregulation of AKAP1 induces oxidative stress and mitochondrial fragmentation, that eventually leads to mitophagy and cell death (Schiattarella et al., 2016, 2018). On the contrary, forced relocalization of PKA to mitochondria by AKAP1 reduces mitochondrial stress and prevents oxidative damage and death of cultured neurons isolated from PINK1 knockout mice or subjected to RNAi mediated silencing (Dagda et al., 2011). In this context, the protective effects of AKAP1 are dependent, at least in part, by the potentiation of the NCX3-mediated mitochondrial calcium efflux (Scorziello et al., 2013). This proof-of principle evidence suggests that preventing AKAP1 proteolysis in PD neurons is a potential therapeutic strategy. In this context, small PPI inhibitors, as aptamers or peptidomimetics, that selectively interfere with the Siah2/AKAP1 binding interface or small RNA molecules (siRNAs and miRNAs) targeting Siah2 are expected to selectively inhibit the hypoxia-induced Siah2 axis. Preventing Siah2/AKAP1 complex formation should increase AKAP1 levels and hamper the decline of mitochondrial activities, restoring energy production and survival pathways in PD neurons (Figure 1).

## Concluding Remarks

PD is a neurodegenerative disorder that affects millions of people worldwide and it is characterized by a progressive motor impairment often associated with other major neurological defects, including dementia. Current therapeutic strategies mostly rely on the use of dopamine agonists and drugs that alleviate the central and peripheral neurological symptoms. However, to be effective, the treatment must start even before the appearance of the neurological signs. Important progress has been made to improve early diagnosis and to identify the relevant pathogenetic factors and mechanisms involved in the selective loss of dopaminergic neurons. However, the causes of most cases of PD are still unknown. Derangement of mitochondrial activities is a common pathogenetic aspect of most, if not all, cases of PD. Evidence indicates that deregulation of mitochondrial homeostasis represents an important event for the development and progression of PD. Accordingly, restoring dysfunctional mitochondrial in PD neurons has been proved to be effective in preventing oxidative stress and dopaminergic neuronal loss, both in patients-derived cells and in *in vivo* models. Understanding the biology of mitochondria, dissecting the signaling networks operating on mitochondria, and identifying the mechanisms responsible for oxidative damage and loss of dopaminergic neurons will undoubtedly contribute to molecular-tailored therapies of PD.

## Author Contributions

AF conceptualized and wrote the manuscript with contributions from DB, AS, MS, RD, and MM.

## Conflict of Interest

The authors declare that the research was conducted in the absence of any commercial or financial relationships that could be construed as a potential conflict of interest.

## References

[B1] Ahuja L. G. Taylor S. S. Kornev A. P. (2019). Tuning the “violin” of protein kinases: the role of dynamics-based allostery. IUBMB Life 71, 685–696. 10.1002/iub.2057 31063633 PMC6690483

[B2] Anderson A. J. Jackson T. D. Stroud D. A. Stojanovski D. (2019). Mitochondria-hubs for regulating cellular biochemistry: emerging concepts and networks. Open Biol. 9:190126. 10.1098/rsob.190126 31387448 PMC6731593

[B3] Baughman J. M. Nilsson R. Gohil V. M. Arlow D. H. Gauhar Z. Mootha V. K. (2009). A computational screen for regulators of oxidative phosphorylation implicates SLIRP in mitochondrial RNA homeostasis. PLoS Genet. 5:e1000590. 10.1371/journal.pgen.1000590 19680543 PMC2721412

[B4] Bingol B. Tea J. S. Phu L. Reichelt M. Bakalarski C. E. Song Q. H.. (2014). The mitochondrial deubiquitinase USP30 opposes parkin-mediated mitophagy. Nature 510, 370–375. 10.1038/nature13418 24896179

[B5] Bose A. Beal M. F. (2016). Mitochondrial dysfunction in Parkinson’s disease. J. Neurochem. 139, 216–231. 10.1111/jnc.13731 27546335

[B6] Boyman L. Karbowski M. Lederer W. J. (2020). Regulation of mitochondrial ATP production: Ca^2+^ signaling and quality control. Trends Mol. Med. 26, 21–39. 10.1016/j.molmed.2019.10.007 31767352 PMC7921598

[B7] Burbulla L. F. Song P. Mazzulli J. R. Zampese E. Wong Y. C. Jeon S.. (2017). Dopamine oxidation mediates mitochondrial and lysosomal dysfunction in Parkinson’s disease. Science 357, 1255–1261. 10.1126/science.aam9080 28882997 PMC6021018

[B8] Caggiu E. Arru G. Hosseini S. Niegowska M. Sechi G. Zarbo I. R.. (2019). Inflammation, infectious triggers, and Parkinson’s disease. Front. Neurol. 10:122. 10.3389/fneur.2019.00122 30837941 PMC6389614

[B9] Cali T. Ottolini D. Brini M. (2011). Mitochondria, calcium, and endoplasmic reticulum stress in Parkinson’s disease. Biofactors 37, 228–240. 10.1002/biof.159 21674642

[B10] Cali T. Ottolini D. Brini M. (2012). Mitochondrial Ca^2+^ as a key regulator of mitochondrial activities. Adv. Exp. Med. Biol. 942, 53–73. 10.1007/978-94-007-2869-1_3 22399418

[B11] Cali T. Ottolini D. Brini M. (2013). Calcium and endoplasmic reticulum-mitochondria tethering in neurodegeneration. DNA Cell Biol. 32, 140–146. 10.1089/dna.2013.2011 23477673

[B12] Carlucci A. Adornetto A. Scorziello A. Viggiano D. Foca M. Cuomo O.. (2008a). Proteolysis of AKAP121 regulates mitochondrial activity during cellular hypoxia and brain ischaemia. EMBO J. 27, 1073–1084. 10.1038/emboj.2008.33 18323779 PMC2323260

[B13] Carlucci A. Lignitto L. Feliciello A. (2008b). Control of mitochondria dynamics and oxidative metabolism by cAMP, AKAPs and the proteasome. Trends Cell Biol. 18, 604–613. 10.1016/j.tcb.2008.09.006 18951795

[B14] Castaldo P. Cataldi M. Magi S. Lariccia V. Arcangeli S. Amoroso S. (2009). Role of the mitochondrial sodium/calcium exchanger in neuronal physiology and in the pathogenesis of neurological diseases. Prog. Neurobiol. 87, 58–79. 10.1016/j.pneurobio.2008.09.017 18952141

[B16] Chan D. C. (2020). Mitochondrial dynamics and its involvement in disease. Annu. Rev. Pathol. 15, 235–259. 10.1146/annurev-pathmechdis-012419-032711 31585519

[B15] Chan C. S. Guzman J. N. Ilijic E. Mercer J. N. Rick C. Tkatch T.. (2007). ‘Rejuvenation’ protects neurons in mouse models of Parkinson’s disease. Nature 447, 1081–1086. 10.1038/nature05865 17558391

[B17] Chen G. Han Z. Feng D. Chen Y. F. Chen L. B. Wu H.. (2014). A regulatory signaling loop comprising the PGAM5 phosphatase and CK2 controls receptor-mediated mitophagy. Mol. Cell 54, 362–377. 10.1016/j.molcel.2014.02.034 24746696

[B18] Dagda R. K. Gusdon A. M. Pien I. Strack S. Green S. Li C.. (2011). Mitochondrially localized PKA reverses mitochondrial pathology and dysfunction in a cellular model of Parkinson’s disease. Cell Death Differ. 18, 1914–1923. 10.1038/cdd.2011.74 21637291 PMC3177020

[B19] Danzer K. M. Haasen D. Karow A. R. Moussaud S. Habeck M. Giese A.. (2007). Different species of α-synuclein oligomers induce calcium influx and seeding. J. Neurosci. 27, 9220–9232. 10.1523/JNEUROSCI.2617-07.2007 17715357 PMC6672196

[B20] Das Banerjee T. Dagda R. Y. Dagda M. Chu C. T. Rice M. Vazquez-Mayorga E.. (2017). PINK1 regulates mitochondrial trafficking in dendrites of cortical neurons through mitochondrial PKA. J. Neurochem. 142, 545–559. 10.1111/jnc.14083 28556983 PMC5554084

[B21] De Stefani D. Raffaello A. Teardo E. Szabo I. Rizzuto R. (2011). A forty-kilodalton protein of the inner membrane is the mitochondrial calcium uniporter. Nature 476, 336–340. 10.1038/nature10230 21685888 PMC4141877

[B22] Decressac M. Mattsson B. Weikop P. Lundblad M. Jakobsson J. Bjorklund A. (2013). TFEB-mediated autophagy rescues midbrain dopamine neurons from α-synuclein toxicity. Proc. Natl. Acad. Sci. U S A 110, E1817–E1826. 10.1073/pnas.1305623110 23610405 PMC3651458

[B23] Dudek J. (2017). Role of cardiolipin in mitochondrial signaling pathways. Front. Cell Dev. Biol. 5:90. 10.3389/fcell.2017.00090 29034233 PMC5626828

[B24] Dukic A. R. McClymont D. W. Taskén K. (2017). A cell-based high-throughput assay for gap junction communication suitable for assessing connexin 43-ezrin interaction disruptors using IncuCyte ZOOM. SLAS Discov. 22, 77–85. 10.1177/1087057116669120 27628689

[B25] Ejlerskov P. Hultberg J. G. Wang J. Carlsson R. Ambjorn M. Kuss M.. (2015). Lack of neuronal IFN-β-IFNAR causes lewy body- and Parkinson’s disease-like dementia. Cell 163, 324–339. 10.1016/j.cell.2015.08.069 26451483 PMC4601085

[B26] Feliciello A. Gottesman M. E. Avvedimento E. V. (2001). The biological functions of A-kinase anchor proteins. J. Mol. Biol. 308, 99–114. 10.1006/jmbi.2001.4585 11327755

[B27] Funayama M. Ohe K. Amo T. Furuya N. Yamaguchi J. Saiki S.. (2015). CHCHD2 mutations in autosomal dominant late-onset Parkinson’s disease: a genome-wide linkage and sequencing study. Lancet Neurol. 14, 274–282. 10.1016/S1474-4422(14)70266-2 25662902

[B28] Gandhi S. Wood-Kaczmar A. Yao Z. Plun-Favreau H. Deas E. Klupsch K.. (2009). PINK1-associated Parkinson’s disease is caused by neuronal vulnerability to calcium-induced cell death. Mol. Cell 33, 627–638. 10.1016/j.molcel.2009.02.013 19285945 PMC2724101

[B29] Gan-Or Z. Dion P. A. Rouleau G. A. (2015). Genetic perspective on the role of the autophagy-lysosome pathway in Parkinson disease. Autophagy 11, 1443–1457. 10.1080/15548627.2015.1067364 26207393 PMC4590678

[B30] Grace A. A. Bunney B. S. (1983). Intracellular and extracellular electrophysiology of nigral dopaminergic neurons—1. Neuroscience 10, 301–315. 10.1016/0306-4522(83)90135-5 6633863

[B970] Grassi D. Howard S. Zhou M. Diaz-Perez N. Urban N. T. Guerrero-Given D.. (2018). Identification of a highly neurotoxic α-synyclein species inducing mitochondrial damage and mitophagy in Parkinson’s disease. Proc. Natl. Acad. Sci. U S A 115, E2634–E2643. 10.1073/pnas.1713849115 29487216 PMC5856519

[B31] Guzman J. N. Sanchez-Padilla J. Wokosin D. Kondapalli J. Ilijic E. Schumacker P. T.. (2010). Oxidant stress evoked by pacemaking in dopaminergic neurons is attenuated by DJ-1. Nature 468, 696–700. 10.1038/nature09536 21068725 PMC4465557

[B32] Harper J. W. Ordureau A. Heo J. M. (2018). Building and decoding ubiquitin chains for mitophagy. Nat. Rev. Mol. Cell Biol. 19, 93–108. 10.1038/nrm.2017.129 29358684

[B33] Hasegawa K. Yasuda T. Shiraishi C. Fujiwara K. Przedborski S. Mochizuki H.. (2016). Promotion of mitochondrial biogenesis by necdin protects neurons against mitochondrial insults. Nat. Commun. 7:10943. 10.1038/ncomms10943 26971449 PMC4793078

[B34] Herst P. M. Rowe M. R. Carson G. M. Berridge M. V. (2017). Functional mitochondria in health and disease. Front. Endocrinol. Lausanne. 8:296. 10.3389/fendo.2017.00296 29163365 PMC5675848

[B35] Hou Y. J. Dan X. L. Babbar M. Wei Y. Hasselbalch S. G. Croteau D. L.. (2019). Ageing as a risk factor for neurodegenerative disease. Nat. Rev. Neurol. 15, 565–581. 10.1038/s41582-019-0244-7 31501588

[B37] Huang E. Qu D. Huang T. Rizzi N. Boonying W. Krolak D.. (2017). PINK1-mediated phosphorylation of LETM1 regulates mitochondrial calcium transport and protects neurons against mitochondrial stress. Nat. Commun. 8:1399. 10.1038/s41467-017-01435-1 29123128 PMC5680261

[B38] Iannielli A. Bido S. Folladori L. Segnali A. Cancellieri C. Maresca A.. (2018). Pharmacological inhibition of necroptosis protects from dopaminergic neuronal cell death in Parkinson’s disease models. Cell Rep. 22, 2066–2079. 10.1016/j.celrep.2018.01.089 29466734 PMC5842028

[B39] Imai Y. (2020). PINK1-Parkin signaling in Parkinson’s disease: lessons from *Drosophila*. Neurosci. Res. [Epub ahead of print]. 10.1016/j.neures.2020.01.016 32035987

[B40] Inoue N. Ogura S. Kasai A. Nakazawa T. Ikeda K. Higashi S. A.. (2018). Knockdown of the mitochondria-localized protein p13 protects against experimental parkinsonism. EMBO Rep. 19:e44860. 10.15252/embr.201744860 29371327 PMC5836091

[B41] Jiang X. Li L. Ying Z. X. Pan C. J. Huang S. Q. Li L.. (2016). A small molecule that protects the integrity of the electron transfer chain blocks the mitochondrial apoptotic pathway. Mol. Cell 63, 229–239. 10.1016/j.molcel.2016.06.016 27447985

[B42] Kennedy E. J. Scott J. D. (2015). Selective disruption of the AKAP signaling complexes. Methods Mol. Biol. 1294, 137–150. 10.1007/978-1-4939-2537-7_11 25783883 PMC4722817

[B43] Kostic M. Ludtmann M. H. R. Bading H. Hershfinke M. Steer E. Chu C. T.. (2015). PKA phosphorylation of NCLX reverses mitochondria! calcium overload and depolarization, promoting survival of PINK1-deficient dopaminergic neurons. Cell Rep. 13, 376–386. 10.1016/j.celrep.2015.08.079 26440884 PMC4709126

[B44] Koyano F. Yamano K. Kosako H. Kimura Y. Kimura M. Fujiki Y.. (2019). Parkin-mediated ubiquitylation redistributes MITOL/March5 from mitochondria to peroxisomes. EMBO Rep. 20:e47728. 10.15252/embr.201947728 31602805 PMC6893362

[B45] Kumar V. (2019). Toll-like receptors in the pathogenesis of neuroinflammation. J. Neuroimmunol 332, 16–30. 10.1016/j.jneuroim.2019.03.012 30928868

[B46] Labzin L. I. Heneka M. T. Latz E. (2018). Innate immunity and neurodegeneration. Annu. Rev. Med. 69, 437–449. 10.1146/annurev-med-050715-104343 29106805

[B47] Lenhausen A. M. Wilkinson A. S. Lewis E. M. Dailey K. M. Scott A. J. Khan S.. (2016). Apoptosis inducing factor binding protein PGAM5 triggers mitophagic cell death that is inhibited by the ubiquitin ligase activity of X-linked inhibitor of apoptosis. Biochemistry 55, 3285–3302. 10.1021/acs.biochem.6b00306 27218139

[B48] Lin G. Lee P. T. Chen K. Mao D. Tan K. L. Zuo Z.. (2018). Phospholipase PLA2G6, a parkinsonism-associated gene, affects Vps26 and Vps35, retromer function and ceramide levels, similar to α-synuclein gain. Cell Metab. 28, 605.e6–618.e6. 10.1016/j.cmet.2018.05.019 29909971

[B49] Lin K. J. Lin K. L. Chen S. D. Liou C. W. Chuang Y. C. Lin H. Y.. (2019). The overcrowded crossroads: mitochondria, α-synuclein and the endo-lysosomal system interaction in Parkinson’s disease. Int. J. Mol. Sci. 20:E5312. 10.3390/ijms20215312 31731450 PMC6862467

[B50] Lu W. Karuppagounder S. S. Springer D. A. Allen M. D. Zheng L. X. Chao B.. (2014). Genetic deficiency of the mitochondrial protein PGAM5 causes a Parkinson’s-like movement disorder. Nat. Commun. 5:4930. 10.1038/ncomms5930 25222142 PMC4457367

[B51] Ludtmann M. H. R. Angelova P. R. Horrocks M. H. Choi M. L. Rodrigues M. Baev A. Y.. (2018). α-synuclein oligomers interact with ATP synthase and open the permeability transition pore in Parkinson’s disease. Nat. Commun. 9:2293. 10.1038/s41467-018-04422-2 29895861 PMC5997668

[B52] Martins R. D. Glaser V. Aguiar A. S. Ferreira P. M. D. Ghisoni K. Scheffer D. D.. (2018). *De novo* tetrahydrobiopterin biosynthesis is impaired in the inflammed striatum of Parkin^−/−^ mice. Cell Biol. Int. 42, 725–733. 10.1002/cbin.10969 29624777

[B53] Matheoud D. Cannon T. Voisin A. Penttinen A. M. Ramet L. Fahmy A. M.. (2019). Intestinal infection triggers Parkinson’s disease-like symptoms in *Pink1*^−/−^ mice. Nature 571, 565–569. 10.1038/s41586-019-1405-y 31316206

[B54] Matheoud D. Sugiura A. Bellemare-Pelletier A. Laplante A. Rondeau C. Chemali M.. (2016). Parkinson’s disease-related proteins PINK1 and Parkin repress mitochondrial antigen presentation. Cell 166, 314–327. 10.1016/j.cell.2016.05.039 27345367

[B55] McConnell B. K. Popovic Z. Mal N. Lee K. Bautista J. Forudi F.. (2009). Disruption of protein kinase A interaction with A-kinase-anchoring proteins in the heart *in vivo*: effects on cardiac contractility, protein kinase A phosphorylation, and troponin I proteolysis. J. Biol. Chem. 284, 1583–1592. 10.1074/jbc.M806321200 18945669 PMC2615525

[B56] McLelland G. L. Soubannier V. Chen C. X. McBride H. M. Fon E. A. (2014). Parkin and PINK1 function in a vesicular trafficking pathway regulating mitochondrial quality control. EMBO J. 33, 282–295. 10.1002/embj.201385902 24446486 PMC3989637

[B57] Meng H. Yamashita C. Shiba-Fukushima K. Inoshita T. Funayama M. Sato S.. (2017). Loss of Parkinson’s disease-associated protein CHCHD2 affects mitochondrial crista structure and destabilizes cytochrome c. Nat. Commun. 8:15500. 10.1038/ncomms15500 28589937 PMC5467237

[B58] Merrill R. A. Strack S. (2014). Mitochondria: a kinase anchoring protein 1, a signaling platform for mitochondrial form and function. Int. J. Biochem. Cell Biol. 48, 92–96. 10.1016/j.biocel.2013.12.012 24412345 PMC3940257

[B59] Moors T. E. Hoozemans J. J. Ingrassia A. Beccari T. Parnetti L. Chartier-Harlin M. C.. (2017). Therapeutic potential of autophagy-enhancing agents in Parkinson’s disease. Mol. Neurodegener. 12:11. 10.1186/s13024-017-0154-3 28122627 PMC5267440

[B60] Muda K. Bertinetti D. Gesellchen F. Hermann J. S. von Zweydorf F. Geerlof A.. (2014). Parkinson-related LRRK2 mutation R1441C/G/H impairs PKA phosphorylation of LRRK2 and disrupts its interaction with 14–3-3. Proc. Natl. Acad. Sci. U S A 111, E34–E43. 10.1073/pnas.1312701111 24351927 PMC3890784

[B61] Nguyen M. Wong Y. C. Ysselstein D. Severino A. Krainc D. (2019). Synaptic, mitochondrial, and lysosomal dysfunction in Parkinson’s disease. Trends Neurosci. 42, 140–149. 10.1016/j.tins.2018.11.001 30509690 PMC6452863

[B63] Ordureau A. Paulo J. A. Zhang W. Ahfeldt T. Zhang J. C. Cohn E. F.. (2018). Dynamics of PARKIN-dependent mitochondrial ubiquitylation in induced neurons and model systems revealed by digital snapshot proteomics. Mol. Cell 70, 211.e8–227.e8. 10.1016/j.molcel.2018.03.012 29656925 PMC5910199

[B64] Palty R. Hershfinkel M. Sekler I. (2012). Molecular identity and functional properties of the mitochondrial Na^+^/Ca^2+^ exchanger. J. Biol. Chem. 287, 31650–31657. 10.1074/jbc.r112.355867 22822063 PMC3442499

[B65] Parihar M. S. Parihar A. Fujita M. Hashimoto M. Ghafourifar P. (2008). Mitochondrial association of α-synuclein causes oxidative stress. Cell. Mol. Life Sci. 65, 1272–1284. 10.1007/s00018-008-7589-1 18322646 PMC11131648

[B66] Parihar M. S. Parihar A. Fujita M. Hashimoto M. Ghafourifar P. (2009). α-synuclein overexpression and aggregation exacerbates impairment of mitochondrial functions by augmenting oxidative stress in human neuroblastoma cells. Int. J. Biochem. Cell Biol. 41, 2015–2024. 10.1016/j.biocel.2009.05.008 19460457

[B67] Parisiadou L. Yu J. Sgobio C. Xie C. Liu G. Sun L.. (2014). LRRK2 regulates synaptogenesis and dopamine receptor activation through modulation of PKA activity. Nat. Neurosci. 17, 367–376. 10.1038/nn.3636 24464040 PMC3989289

[B68] Poenisch M. Burger N. Staeheli P. Bauer G. Schneider U. (2009). Protein X of borna disease virus inhibits apoptosis and promotes viral persistence in the central nervous systems of newborn-infected rats. J. Virol. 83, 4297–4307. 10.1128/jvi.02321-08 19211764 PMC2668499

[B69] Pryde K. R. Smith H. L. Chau K. Y. Schapira A. H. (2016). PINK1 disables the anti-fission machinery to segregate damaged mitochondria for mitophagy. J. Cell Biol. 213, 163–171. 10.1083/jcb.201509003 27091447 PMC5084273

[B70] Przedborski S. (2017). The two-century journey of Parkinson disease research. Nat. Rev. Neurosci. 18, 251–259. 10.1038/nrn.2017.25 28303016

[B71] Ren Q. Ma M. Yang J. Nonaka R. Yamaguchi A. Ishikawa K.. (2018). Soluble epoxide hydrolase plays a key role in the pathogenesis of Parkinson’s disease. Proc. Natl. Acad. Sci. U S A 115, E5815–E5823. 10.1073/pnas.1802179115 29735655 PMC6016799

[B72] Rinaldi L. Delle Donne R. Borzacchiello D. Insabato L. Feliciello A. (2018). The role of compartmentalized signaling pathways in the control of mitochondrial activities in cancer cells. Biochim. Biophys. Acta Rev. Cancer 1869, 293–302. 10.1016/j.bbcan.2018.04.004 29673970

[B73] Rinaldi L. Delle Donne R. Sepe M. Porpora M. Garbi C. Chiuso F.. (2016). praja2 regulates KSR1 stability and mitogenic signaling. Cell Death Dis. 7:e2230. 10.1038/cddis.2016.109 27195677 PMC4917648

[B74] Rinaldi L. Sepe M. Donne R. D. Feliciello A. (2015). A dynamic interface between ubiquitylation and cAMP signaling. Front. Pharmacol. 6:177. 10.3389/fphar.2015.00177 26388770 PMC4559665

[B75] Rinaldi L. Sepe M. Delle Donne R. Conte K. Arcella A. Borzacchiello D.. (2017). Mitochondrial AKAP1 supports mTOR pathway and tumor growth. Cell Death Dis. 8:e2842. 10.1038/cddis.2017.241 28569781 PMC5520900

[B76] Rocha E. M. Smith G. A. Park E. Cao H. Brown E. Hallett P.. (2015). Progressive decline of glucocerebrosidase in aging and Parkinson’s disease. Ann. Clin. Transl. Neurol. 2, 433–438. 10.1002/acn3.177 25909088 PMC4402088

[B77] Ryan T. Bamm V. V. Stykel M. G. Coackley C. L. Humphries K. M. Jamieson-Williams R.. (2018). Cardiolipin exposure on the outer mitochondrial membrane modulates α-synuclein. Nat. Commun. 9:817. 10.1038/s41467-018-03241-9 29483518 PMC5827019

[B78] Schiattarella G. G. Boccella N. Paolillo R. Cattaneo F. Trimarco V. Franzone A.. (2018). Loss of *Akap1* exacerbates pressure overload-induced cardiac hypertrophy and heart failure. Front. Physiol. 9:558. 10.3389/fphys.2018.00558 29892230 PMC5985454

[B79] Schiattarella G. G. Cattaneo F. Pironti G. Magliulo F. Carotenuto G. Pirozzi M.. (2016). Akap1 deficiency promotes mitochondrial aberrations and exacerbates cardiac injury following permanent coronary ligation *via* enhanced mitophagy and apoptosis. PLoS One 11:e0158934. 10.1371/journal.pone.0158934 27136357 PMC4852950

[B80] Schmidt H. (2012). Three functional facets of calbindin D-28k. Front. Mol. Neurosci. 5:25. 10.3389/fnmol.2012.00025 22435048 PMC3304297

[B81] Scorziello A. Savoia C. Sisalli M. J. Adornetto A. Secondo A. Boscia F.. (2013). NCX3 regulates mitochondrial Ca^2+^ handling through the AKAP121-anchored signaling complex and prevents hypoxia-induced neuronal death. J. Cell Sci. 126, 5566–5577. 10.1242/jcs.129668 24101730

[B82] Sirabella R. Sisalli M. J. Costa G. Omura K. Ianniello G. Pinna A.. (2018). NCX1 and NCX3 as potential factors contributing to neurodegeneration and neuroinflammation in the A53T transgenic mouse model of Parkinson’s disease. Cell Death Dis. 9:725. 10.1038/s41419-018-0775-7 29941946 PMC6018508

[B83] Sliter D. A. Martinez J. Hao L. Chen X. Sun N. Fischer T. D.. (2018). Parkin and PINK1 mitigate STING-induced inflammation. Nature 561, 258–262. 10.1038/s41586-018-0448-9 30135585 PMC7362342

[B84] Sorrentino V. Menzies K. J. Auwerx J. (2018). Repairing mitochondrial dysfunction in disease. Annu. Rev. Pharmacol. Toxicol. 58, 353–389. 10.1146/annurev-pharmtox-010716-104908 28961065

[B85] Surmeier D. J. Schumacker P. T. (2013). Calcium, bioenergetics, and neuronal vulnerability in Parkinson’s disease. J. Biol. Chem. 288, 10736–10741. 10.1074/jbc.r112.410530 23086948 PMC3624453

[B86] Surmeier D. J. Guzman J. N. Sanchez-Padilla J. Schumacker P. T. (2011). The role of calcium and mitochondrial oxidant stress in the loss of substantia nigra pars compacta dopaminergic neurons in Parkinson’s disease. Neuroscience 198, 221–231. 10.1016/j.neuroscience.2011.08.045 21884755 PMC3244353

[B87] Szelechowski M. Betourné A. Monnet Y. Ferré C. A. Thouard A. Foret C.. (2014). A viral peptide that targets mitochondria protects against neuronal degeneration in models of Parkinson’s disease. Nat. Commun. 5:5181. 10.1038/ncomms6181 25333748

[B88] Tan W. Z. Colombini M. (2007). VDAC closure increases calcium ion flux. Biochim. Biophys. Acta 1768, 2510–2515. 10.1016/j.bbamem.2007.06.002 17617374 PMC2220155

[B89] Tanaka A. Cleland M. M. Xu S. Narendra D. P. Suen D. F. Karbowski M.. (2010). Proteasome and p97 mediate mitophagy and degradation of mitofusins induced by Parkin. J. Cell Biol. 191, 1367–1380. 10.1083/jcb.201007013 21173115 PMC3010068

[B90] Tolosa E. Vila M. Klein C. Rascol O. (2020). LRRK2 in Parkinson disease: challenges of clinical trials. Nat. Rev. Neurol. 16, 97–107. 10.1038/s41582-019-0301-2 31980808

[B91] Tsigelny I. F. Sharikov Y. Wrasidlo W. Gonzalez T. Desplats P. A. Crews L.. (2012). Role of α-synuclein penetration into the membrane in the mechanisms of oligomer pore formation. FEBS J. 279, 1000–1013. 10.1111/j.1742-4658.2012.08489.x 22251432 PMC3925782

[B92] Verma M. Callio J. Otero P. A. Sekler I. Wills Z. P. Chu C. T. (2017). Mitochondrial calcium dysregulation contributes to dendrite degeneration mediated by PD/LBD-associated LRRK2 mutants. J. Neurosci. 37, 11151–11165. 10.1523/JNEUROSCI.3791-16.2017 29038245 PMC5688524

[B93] Verstraeten A. Theuns J. Van Broeckhoven C. (2015). Progress in unraveling the genetic etiology of Parkinson disease in a genomic era. Trends Genet 31, 140–149. 10.1016/j.tig.2015.01.004 25703649

[B94] Wang L. M. Cho Y. L. Tang Y. C. Wang J. G. Park J. E. Wu Y. J.. (2018). PTEN-L is a novel protein phosphatase for ubiquitin dephosphorylation to inhibit PINK1-Parkin-mediated mitophagy. Cell Res. 28, 872–873. 10.1038/s41422-018-0056-0 30038297 PMC6082851

[B96] Wang W. T. Pan G. Q. Zhang Z. Y. Suo Z. W. Yang X. Hu X. D. (2015). Ht31 peptide inhibited inflammatory pain by blocking NMDA receptor-mediated nociceptive transmission in spinal dorsal horn of mice. Neuropharmacology 89, 290–297. 10.1016/j.neuropharm.2014.09.031 25312281

[B95] Wang L. Qi H. Tang Y. Shen H. M. (2020). Post-translational modifications of key machinery in the control of mitophagy. Trends Biochem. Sci. 45, 58–75. 10.1016/j.tibs.2019.08.002 31606339

[B97] Wang W. Wang X. Fujioka H. Hoppel C. Whone A. L. Caldwell M. A.. (2016). Parkinson’s disease-associated mutant VPS35 causes mitochondrial dysfunction by recycling DLP1 complexes. Nat. Med. 22, 54–63. 10.1038/nm.3983 26618722 PMC4826611

[B98] Wang X. Winter D. Ashrafi G. Schlehe J. Wong Y. L. Selkoe D.. (2011). PINK1 and Parkin target Miro for phosphorylation and degradation to arrest mitochondrial motility. Cell 147, 893–906. 10.1016/j.cell.2011.10.018 22078885 PMC3261796

[B99] Wang Z. G. Jiang H. Chen S. Du F. H. Wang X. D. (2012). The mitochondrial phosphatase PGAM5 functions at the convergence point of multiple necrotic death pathways. Cell 148, 228–243. 10.1016/j.cell.2011.11.030 22265414

[B100] Wauters F. Cornelissen T. Imberechts D. Martin S. Koentjoro B. Sue C.. (2019). *LRRK2* mutations impair depolarization-induced mitophagy through inhibition of mitochondrial accumulation of RAB10. Autophagy 16, 203–222. 10.1080/15548627.2019.1603548 30945962 PMC6984591

[B101] Weihofen A. Thomas K. J. Ostaszewski B. L. Cookson M. R. Selkoe D. J. (2009). Pink1 forms a multiprotein complex with Miro and Milton, linking Pink1 function to mitochondrial trafficking. Biochemistry 48, 2045–2052. 10.1021/bi8019178 19152501 PMC2693257

[B102] Wood-Kaczmar A. Deas E. Wood N. W. Abramov A. Y. (2013). The role of the mitochondrial NCX in the mechanism of neurodegeneration in Parkinson’s disease. Adv. Exp. Med. Biol. 961, 241–249. 10.1007/978-1-4614-4756-6_20 23224884

